# Network analysis of empathy, anxiety and depression symptoms, relationship satisfaction, sensory processing sensitivity, and alexithymia

**DOI:** 10.1038/s41598-025-24677-2

**Published:** 2025-11-20

**Authors:** Lukas Novak, Klara Malinakova, Kateřina Juklová, Josef Koláček, Radka Zidkova, Zdenek Meier, Peter Tavel, Jitse P. van Dijk, Andrea F. de Winter

**Affiliations:** 1 Olomouc University Social Health Institute, Palacký University, Olomouc, Czech Republic; 2 Department of Community and Occupational Medicine, University Medical Center Groningen, University of Groningen, Groningen, The Netherlands; 3 Graduate School Kosice Institute for Society and Health, P.J. Safarik University in Kosice, Kosice, Slovak Republic; 4 Department of Pedagogy and Psychology, Faculty of Education, University of Hradec Kralove, Hradec Kralove, Czech Republic

**Keywords:** Psychology, Psychiatric disorders

## Abstract

Mood disorders, like anxiety and depression, are the most prevalent mental health issues that significantly impact both individuals and society. Thus, the exploration of risk and protective factors that may influence anxiety and depression symptoms is of great importance. Studies have shown that higher emotional empathy is often linked to increased anxiety and depression, yet results are mixed. Understanding this link in more depth may be relevant for psychotherapists, as it may help them in creating effective treatment plans for anxiety and depression. Therefore, this study aims to explore how positive and negative emotional empathy are linked with anxiety/depression symptoms once relationship satisfaction, sensory‑processing sensitivity (SPS), and alexithymia are taken into account. In 3 382 Czech adults (mean age = 31.76 ± 13.13; 66.29% Females), we administered the Overall Anxiety Severity and Impairment Scale (OASIS), Overall Depression Severity and Impairment Scale (ODSIS), Toronto Empathy Questionnaire (TEQ), Kansas Marital Satisfaction Scale (KMSS), Sensory‑Processing Sensitivity Questionnaire – SPSQ – (sensory subscale), and Perth Alexithymia Questionnaire – PAQ – (externally‑oriented‑thinking sub‑scale). Mixed Graphical Model networks were estimated for (a) total scores and (b) individual items, age, gender, and education were controlled. Accuracy and stability were explored via non‑parametric bootstrapping. At the total‑score level, higher emotional empathy was positively linked with anxiety and depression. Both links weakened after covariates were entered. Empathy nevertheless retained positive edges with SPS and relationship satisfaction, and a negative edge with alexithymia. In the item‑level network, no negative‑empathy items related to anxiety or depression. One positive‑empathy item (“When someone close to me is happy, it affects me deeply - in a positive way”) showed an indirect association with lower anxiety via greater relationship satisfaction. Bootstrapping indicated acceptable stability for centrality indices but wide CIs for some edge weights. Our findings suggest that the link between emotional empathy and anxiety/depression is largely indirect. Positive empathy may buffer against anxiety via higher relationship satisfaction. Deconstructing these constructs to the item level may provide more specific insights than analyzing total scores alone. Future empathy researchers should also use longitudinal designs to examine potential causal relationships between empathy and anxiety/depression.

## Introduction

Mood disorders, like anxiety and depression, are the most prevalent mental health issues that have a profound impact both on individual lives and on society as a whole^1–3^. In terms of social impact, they represent the highest financial burden among all mental health disorders^4^. In the United States, for instance, the economic burden linked with these disorders reached 46.6 billion dollars per year^4^, which represents almost 1% of the annual Gross Domestic Product (GDP). Similarly, in the United Kingdom, the economic burden associated with anxiety disorders was around 0.68% of the annual GDP^5^. Besides these economic costs for society, anxiety and depressive disorders are linked with other health issues that pose a substantial burden for people suffering from them.

Anxiety and depressive disorders are related to substance abuse^6^, lower quality of life^7^, sleep problems^8^, cognitive deficits^9^, and suicide^10^. Moreover, people with these disorders are also more likely to have physical health problems such as cancer^11^, gastrointestinal diseases^11^, cardiovascular disease^12^, premature mortality^12^, migraine, arthritis, back pain, and other physical health issues^13^. In addition, recent research suggested that the number of people suffering from anxiety/depression tends to increase over time^14,15^.

It was estimated that the number of persons affected by these disorders rose by 55% from 1990 to 2019^16^. This increase was especially pronounced in countries with a high socio-demographic index^17^. Furthermore, the COVID-19 pandemic further accelerated the rise in anxiety and depressive disorders^18^. Taken together, anxiety and depression are the most common psychiatric illnesses. It is, therefore, essential to understand which factors might contribute to higher levels of their symptoms. One of these factors with growing research attention is empathy.

Although there are many different conceptualizations of empathy^19^, in general, two types can be distinguished: cognitive and emotional empathy. Cognitive empathy represents the capacity to estimate and predict the mental states of others^20^. Emotional empathy is characterized by understanding and sharing the emotions of others^21^. According to Morelli et al.^21^, in emotional empathy, we can further distinguish two different aspects, i.e., positive and negative empathy. Whereas positive emotional empathy represents the capacity to understand and share the positive emotions of another person (e.g., joy), negative emotional empathy represents the same capacity but it is directed towards negative emotions (e.g., distress). Thus, the distinction between these two aspects of empathy is based on the type of emotions that are shared—either positive or negative.

So far, two key meta-analyses have been published: one explores the link between emotional empathy and depression^22^, and the other examines the connection between emotional empathy and anxiety^23^. Both of these meta-analyses report mixed results, and their authors conclude that one of the key reasons why the link between emotional empathy and these mental health conditions is heterogeneous is that emotional empathy itself is a heterogeneous construct. For instance, in studies included in these meta-analyses, some aspects of emotional empathy (e.g., empathic distress) were rather positively associated with both disorders. In contrast, some other aspects of emotional empathy (e.g., empathic concern) were negligibly linked to anxiety/depression. Thus, the authors of both meta-analyses suggest that future researchers should distinguish between the various aspects of emotional empathy, as each may have a different relationship with these conditions. However, to the best of our knowledge, no research has looked at how positive and negative empathy together relate to anxiety or depression.

However, modeling the link between positive or negative empathy and anxiety/depression might be challenging. It is theoretically plausible that the associations between emotional empathy and these two mental health conditions are complex: distinct aspects of emotional empathy can be related to anxiety/depression in various ways due to the effect of different variables. To better understand these intricate relationships, there is a need to use statistical techniques that would, at least partially, allow us to capture such a degree of complexity. One of these statistical techniques is network analysis – a multivariate method that allows for the examination of direct and indirect relationships between variables^24^. To investigate the links between emotional empathy and mental health issues, it would be important to include variables that might influence these links. These variables might include sensory processing sensitivity (SPS), alexithymia, and relationship satisfaction. For a more cohesive explanation of the selection of these constructs, please refer to the Supplementary Material 1 available online.

SPS represents a genetically determined trait involving a deeper processing of stimuli that is, among others, associated with elevated emotional responsiveness^25^. Twin studies indicate that almost half of the variance in SPS is heritable^26^. The meta-analysis of Lionetti et al.^27^ shows that SPS is moderately correlated with neuroticism and negative affect while retaining discriminant validity from Big-Five openness and extraversion. Systematic reviews and meta-analyses further link high SPS to greater vulnerability to anxiety and depressive symptoms in both student and clinical cohorts^28,29^. Longitudinal evidence demonstrates that SPS amplifies both the costs of adverse contexts and the benefits of supportive ones, consistent with Differential-Susceptibility, Environmental Sensitivity, and Vantage-Sensitivity theories of the SPS^30,31^. Neuroimaging work corroborates this heightened responsivity: high-SPS individuals show stronger activation of brain regions involved in awareness, sensory integration, and empathy when viewing others’ emotions^32^. Collectively, these findings support viewing SPS as a marker of environmental sensitivity—partly genetic—that may modulate the pathways between empathy and internalising distress^33,34^.

Alexithymia, characterised by difficulties with identifying and describing feelings and an externally-oriented thinking style, is a well-established trans-diagnostic risk factor for internalising disorders. A recent meta-analysis of 35 studies (*n* = 23,085) confirmed a significant positive association between global alexithymia scores and depression severity^35^. Longitudinal data further show that baseline alexithymia predicts subsequent increases in anxiety and broader psychological distress, and that this effect is partly mediated by emotion-regulation deficits^36,37^. Systematic reviews of social cognition further indicate that alexithymia is consistently linked to reduced emotion recognition and lower empathy^38^.

In relationship satisfaction, empathy could contribute to higher emotional understanding between partners, leading to higher relationship satisfaction. This aligns with meta-analytic evidence suggesting that empathic accuracy and empathic responsiveness are significant determinants of perceived relationship quality^39^. At the same time, longitudinal evidence shows that lower couple satisfaction prospectively predicts the onset and maintenance of depressive and anxiety disorders^40,41^. Taken together, satisfying partnerships appear to foster adaptive emotion-regulation and social-support processes that buffer stress reactivity, whereas dissatisfied relationships magnify the emotional contagion of partners’ distress^42^. Understanding how SPS, alexithymia, and couple satisfaction interact with emotional empathy and anxiety/depression not only enhances our theoretical knowledge but also has practical implications. Through a better understanding of how these variables interact with each other, future studies could develop targeted interventions addressing these specific variables, potentially improving outcomes for individuals dealing with these mental health conditions. Thus, the main aim of the present study is to explore the interplay between empathy, alexithymia, SPS, anxiety/depression, and relationship satisfaction. Based on this aim, the following research question was formulated:

RQ1: When alexithymia, relationship satisfaction, and SPS are modelled together, what direct and indirect associations link emotional empathy with anxiety/depression?

## Methods

### Participants and procedure

The research was conducted through an online questionnaire system (https://dotaznik.oushi.upol.cz/doc/en/). Data gathering occurred between September 2022 and December 2023, utilizing the snowball sampling technique. University students collected the data from adult participants as a component of their educational curriculum and assigned coursework. Before the online questionnaire started, informed consent was obtained from all subjects. All research was performed in accordance with relevant guidelines/regulations. Olomouc University Social Health Institute granted ethical clearance for this study (Approval No. 2020/4).

Out of the initial pool of 7 633 participants, we applied several exclusion criteria to ensure high data quality. Participants were excluded if they: consistently gave identical responses to scale items – meaning that they had zero SD to at least three questionnaires administered (*n* = 277); completed the survey in a very short time (< 26 min; the median survey time was 55.47 min; *n* = 2056); responded inconsistently to control questions regarding age, weight, and height administered at the beginning and end of the survey (*n* = 376); were under 18 years old (*n* = 83); or were suspected of being the same student filling out the survey multiple times to fulfill course requirements (*n* = 1324). Identification of these problematic participants was based on the following two formulas:

$$\:p=\frac{\sum\:_{i=1}^{n}{x}_{ji}\ne\:0}{n}$$

The formula in question calculates the likelihood based on browser compatibility for each participant. In this equation, *p* symbolizes the chance of a match between the browser type and version for each respondent. The term $\:{x}_{ji}$ refers to the count of instances where the i-th item from the student’s code aligns with the j-th item from the browser type and version used for completing the survey; *n* denotes the aggregated count of entries in the matrix. Following this, a threshold was established using these matching probabilities. In the subsequent equation, *m* denotes the total number of surveys a participant completed, and *q* is an identifier assigned to each participant.

$$\:tolerance\:limit=\overline{p}\text{*}med\left({m}_{1},{m}_{2},{m}_{3}...{m}_{k}\right)\text{*}\sum\:_{q=1}^{n}\left({q}_{m}>1\right)$$

In the final step, to account for measurement differences of psychological constructs across diverse language backgrounds, we removed (135) participants who were not of Czech nationality. Thus, the final number of participants was: 3382 (31.76 ± 13.13, range 18 to 80 years; females: 66.29%).

### Measures

### Overall anxiety severity and impairment scale (OASIS)

The OASIS^43^ is a psychological instrument designed to measure how often anxiety symptoms occur, how severe they are, and the effect of anxiety on one´s personal relationships and daily activities at work, school, or home. Second, this tool also measures avoidance of places/objects/activities due to anxiety. The Czech version^44^ contains five items to which respondents should respond on 5-point scale ranging from ‘never’ (0) to ‘all the time’ (4). A higher total score represents higher anxiety. Good psychometric properties of the scale were supported with adequate test retest reliability (*r* = .83) and convergent validity with positive associations to established anxiety measure. Example items include: “How often did you feel anxious?”, “How much has your anxiety affected your social life and relationships?”. In the present study, the OASIS had a Cronbach’s α of 0.92 95% CI [0.91 − 0.92].

### Overall depression severity and impairment scale (ODSIS)

The ODSIS^45^ is a reliable and valid measurement tool assessing the frequency, intensity, and impact of depressive symptoms on interpersonal relationships and everyday tasks in environments such as work, school, or home. The Czech adaptation^44^ of the ODSIS encompasses five questions, and participants are instructed to answer using a 5-point scale that ranges from ‘never’ (0) to ‘all the time’ (4). Good psychometric properties of the scale were supported with adequate test retest reliability (*r* = .85) and convergent validity with positive associations to established depression measure. Example items include: “How often did you feel depressed?”, “How much has your depression affected your social life and relationships?”. A higher score on this scale indicates a higher degree of depression. In the present study, the ODSIS had a Cronbach’s α of 0.97 95% CI [0.97 − 0.97].

### Toronto empathy questionnaire (TEQ)

The TEQ^46^ was designed by combining items from pre-existing empathy scales to create a new measure primarily assessing emotional empathy. The TEQ contains items assessing both positive empathy^47^ and negative empathy^21^. Its Czech version^48^ consists of seven statements on a scale ranging from ‘never’ (0) to ‘always’ (4). A higher total score represents higher emotional empathy. Good psychometric properties of the scale were supported with adequate test retest reliability (*r* = .81) and convergent validity with positive associations to female gender and compassion. Example items include: “I enjoy making other people feel better”, “Other people’s misfortunes do disturb me a great deal”. Higher scores indicate higher degree of emotional empathy. In the present study, the TEQ had a Cronbach’s α of 0.88 95% CI [0.88 − 0.89].

### Perth alexithymia questionnaire (PAQ)

The PAQ was designed by Preece et al. (2018) to measure general alexithymia and its components. The primary components/subscales of alexithymia consist of: (1) generalized difficulty in identifying feelings (G_DIF), (2) generalized difficulty in describing feelings (G_DDF), and (3) generalized externally oriented thinking (G_EOT). Good psychometric properties of the scale were supported with high internal consistency (alpha: 0.94) and convergent validity with positive associations to depression, anxiety and neuroticism^49^. In the present study, only the G_EOT subscale was used because this was the only subscale of the PAQ that was linked with the TEQ score by past research^49^. The G_EOT subscale as included in the Czech version^49^ of the PAQ, consists of eight items on a seven-point Likert scale ranging from ‘strongly disagree’ (1) to ‘strongly agree’ (7). Example items are as follows: “I tend to ignore how I feel”, “It’s not important for me to know what I’m feeling“. Higher score indicates higher degree of externally oriented thinking. In the present study, the G_EOT had a Cronbach’s α of .92 95% CI [0.92 − 0.92].

### Kansas marital satisfaction scale (KMSS)

The KMSS^50^ was initially developed to measure satisfaction in marriage. However, a modified-validated version of the scale^51^ used in the present study assesses satisfaction in romantic relationships, also in non-married couples. This measure comprises the three items on which participants respond using a 7-point Likert scale ranging from ‘extremely dissatisfied’ (1) to ‘extremely satisfied’ (7). A higher score indicates a higher degree of satisfaction with the romantic relationship. Good psychometric properties of the scale were supported with high internal consistency (alpha: 0.92) and convergent validity with positive associations to well-being^51^. Example items included: “How satisfied are you with your counterpart as a partner?”, “How satisfied are you with your relationship with your partner?”. In the present study, the KMSS had a Cronbach’s α of 0.98 95% CI [0.98 − 0.98].

### Sensory processing sensitivity questionnaire (SPSQ)

The SPSQ was developed by Malinakova et al.^52^ to assess the degree to which a person is sensitive to external and internal stimuli. The SPSQ consists of 16 items, individual sensitivity to them is rated on an 11-point scale where participants should indicate the degree to which they are sensitive to stimuli (e.g., light, heat) as compared with others. The response options range from 0 = ‘compared to others, I am not sensitive at all’ to 10 = ‘extremely more sensitive than people around me’. The original psychometric study showed excellent internal consistency (Cronbach’s α = 0.92; McDonald’s ω = 0.92) and very high 2-week test–retest reliability (*r* = 0.95). A bi-factor confirmatory model (CFI = 0.993; TLI = 0.990; RMSEA = 0.070; SRMR = 0.039) supported two correlated factors (subscales): Sensory Sensitivity (SPSQ_S) and Other Sensitivity while measurement invariance tests indicated equivalent functioning across genders^52^. Convergent validity was evidenced by strong associations with the Highly Sensitive Person Scale in the Czech sample (*r* = 0.79) and in an independent Persian validation of the SPSQ (*r* = 0.44) that also replicated good internal consistency: α = 0.83^53^. Criterion-related validity is supported by consistent positive links with neuroticism, anxiety, and depression, and by successful cross-cultural replications of the scale’s structure in a Persian sample^53^. The SPSQ consists of a Sensory processing sensitivity subscale (SPSQ_S), which focuses on sensory stimuli, and the Other Sensitivity subscale (SPSQ_O), consisting of items assessing more general sensitivity to various external and internal stimuli. Items from the SPSQ_S and the SPSQ_O subscales can be summed up to create a single total score (with higher scores meaning a higher degree of the SPS), or the SPSQ_S score can be used separately. In our study, we have chosen the latter option. We used only the SPSQ_S because it focuses specifically on sensory processing without items directly referring to interpersonal sensitivity or emotional responsiveness. This allowed us to minimize conceptual overlap with measures of empathy and avoid tautological associations. In our data, SPSQ_S exhibited good internal consistency with Cronbach’s α of 0.75 95% CI [0.74 − 0.76].

### Statistical analysis

#### Missing data analysis and outlier screening

In the first step of our data screening, we explored the percentage of missing values and missing data patterns. Our investigation revealed that some values in the questionnaires used were missing (OASIS = 7.73%; ODSIS = 7.92%; TEQ = 0.95%; G_EOT = 5.33%; KMSS = 0%, SPSQ_S = 1.48%). The Little Missing Completely At Random (MCAR) test indicated no pattern in missing data. For this reason, missing data points were removed listwise. Finally, we utilized the Median Absolute Deviation (MAD) method to identify outliers. This process revealed that there were 221 outliers across used questionnaires. Examination of these outlying values did not indicate a pattern of uniform responses by participants to items across different questionnaires. For this reason, these participants were not excluded from the dataset.

#### Network analysis

Next, to conduct network analysis, we utilised Markov Random Fields (MRFs). In the MRFs, variables are reflected by nodes (circles), and the relationship between these variables by edges (connections between circles). There are several reasons why we used MRFs instead of traditional statistical techniques. First, each edge in a network is estimated by controlling for all other variables in the network^54^. This provides much more detailed insight into possible causal relationships between variables in a network. Second, the MRFs allow for mapping complex relationships between variables. This means that network analysis can help to detect reciprocal relationships such as feedback loops: e.g., A is related to B, which is backward related with A^55^. Third, in the MRFs, it is possible to examine these complex relationships on the level of individual items. This could help to identify the most central or influential nodes within a network^56^. For instance, in a network of symptoms, some symptoms might be more central and influence many others. Understanding these central elements and their links with other nodes in a network might guide potential treatment/interventions. To identify the most influential variables in a network, it is critical to avoid redundant items. Thus, in the first step of the network estimation, we removed redundant items using the Weighted Topological Overlap method^57^.

Next, we estimated the MRFs using the Mixed Graphical Model (MGM - Haslbeck & Waldorp, 2020). This method was chosen because our data contained mixed types of variables (i.e., ordinal, nominal, and continuous). The MGM performs node-wise network estimation through generalized regression models^59,60^. As every connection in the network is calculated two times (once for each connecting point), an “AND” rule was applied. This means a connection is considered part of the network if it appears in two regression analyses conducted^61^. To decrease the rate of false positive edges, we used the following regularization technique: Least Absolute Shrinkage and Selection Operator (LASSO). The degree of penalty as imposed by LASSO is controlled via the tuning parameter γ, which was set to be 0.5 in this study. Some authors suggest that if this cutoff is used, then network does not contain many false positive edges^59,60^. The best-fitting network model was consequently selected using the Extended Bayesian Information Criterion (EBIC).

In the next step, we estimated node predictability for all variables except the categorical covariates, i.e., education and gender. Mode predictability reflects how much percent of the variance of a given node can be explained by other nodes^62,63^. Next, to quantify the importance of nodes in the network, we estimated the expected influence centrality index. Although more centrality indices exist (such as between-ness or close-ness), we chose to use the expected influence centrality index because of its practical implications: nodes with the highest values of expected influence might be more relevant for treatment/intervention than some other nodes in a network with lower values of expected influence^64^. In order to test the stability of the expected influence index and edge weights, we performed two bootstrapping procedures. First, we performed a non-parametric bootstrap (resampling with replacement) using 2500 samples to explore the stability of edge weights. Second, we tested the stability of the expected influence index by case-dropping subset bootstrap using 2500 samples. After the case-dropping bootstrap procedure was finished, we quantified the stability of the expected influence by correlation stability coefficient (CS-coefficient). Values of this coefficient should range at least above 0.25 and preferably above 0.5^56^.

##### Network types

To explore whether depression and anxiety are differently linked with empathy, we set up two separate networks for analysis: one focused on anxiety and the other on depression. Each network also considered measures of empathy, alexithymia, SPS and relationship satisfaction. Our analyses were carried out in two formats. Initially, we performed a total score analysis, where we used total scores from each measurement instrument. Following this, we conducted an analysis based on individual items, which was the main focus of the present study. Each network was estimated in both a crude and an adjusted form. In the adjusted form, we controlled for variables age, gender, and educational level because well‑documented demographic effects exist for all three. In terms of age, younger adults report higher anxiety^65^ and depression^66^ than older adults. In relation to education, individuals with lower educational attainment exhibit greater risk for depression and anxiety^67^. Empathy is also linked with both age^68^ and gender^48,69^. Controlling for these covariates, therefore, yields edges that more closely represent unique associations among psychological variables of our primary interest. Unless noted otherwise, all substantive interpretations in the Results and Discussion refer to the adjusted networks; the crude graphs are provided for completeness and as a robustness check. Unless stated otherwise, our main conclusions are based on adjusted networks. All analysis were conducted in R^70^ using primary the MGM^58^ and the Psychtoolbox^71^ libraries for statistical analyses.

## Results

### Socio-demographic results

A socio-demographic analysis revealed that most participants were employed, had a high school, and were in a relationship. It also showed significant differences between demographic groups in empathy, anxiety, alexithymia, SPS, and depression scores. Specifically, students had significantly higher depression and anxiety scores compared to both working and unemployed individuals, while unemployed individuals reported significantly higher empathy and lower SPS than students. In terms of education, individuals with just basic school had significantly higher depression, anxiety, and alexithymia scores compared to those with higher education, while also having lower empathy and higher SPS. Married participants had significantly lower depression, anxiety, and SPS scores and higher empathy compared to those not in a relationship. Comparisons between genders revealed that women had significantly higher scores of depression, anxiety, empathy, and SPS, while men had a significantly higher degree of alexithymia. Finally, income was significantly related to anxiety, with lower-income individuals reporting higher levels compared to higher-income earners. Further significant differences are depicted in Table 1.

**Table 1:** Socio-demographic table with means, standard deviations and differences between groups.

variable	*n*(%)	ODSIS Group differences	SPSQ_S Group differences	TEQ Group differences	G_EOT Group differences	OASIS Group differences	TEQ M(SD)	OASIS M(SD)	ODSIS M(SD)	G_EOT M(SD)	SPSQ_S M(SD)
Economic status											
1 Working	1932 (57.13)	*p* < .001 (1–3***, 2–3**)	*p* = .012 (2–3*)	*p* = .003 (1–2**,2–3**)		*p* < .001 (1–3***, 2–3**)	20.92 (4.42)	11.81 (4.35)	9.77 (4.76)	24.84 (10.48)	46.84 (9.21)
2 Unemployed	230 (6.8)						21.71 (4.47)	12.29 (4.62)	10.17 (5.18)	24.2 (10.78)	45.63 (9.36)
3 Student	1220 (36.07)						20.68 (4.63)	13.34 (4.86)	11.48 (5.53)	25.35 (10.93)	47.45 (8.36)
Education											
1 Basic school	178 (5.26)	*p* < .001 (1–2**, 1–3**, 1–4***, 1–5***, 1–6***, 2–5*, 2–6***, 3–5***, 3–6***)		*p* = .001 (1–5***, 1–6**, 3–5**)	*p* < .001 (1–4***, 1–5***, 1–6***, 2–3*, 2–4***, 2–5***, 2–6***, 3–4***, 3–5***, 3–6***)	*p* < .001 (1–2***, 1–3***, 1–4***, 1–5***, 1–6***, 2–6**, 3–5***, 3–6***)	19.81 (4.88)	14.62 (5.19)	12.66 (5.82)	27.57 (10.64)	47.27 (9.32)
2 Secondary vocational school	331 (9.79)						20.67 (5.02)	12.37 (4.3)	10.59 (4.92)	27.43 (9.44)	45.74 (10.13)
3 High school	1744 (51.57)						20.76 (4.6)	12.76 (4.74)	10.94 (5.37)	25.71 (10.78)	47.1 (8.56)
4 Higher vocational school	133 (3.93)						20.81 (4.54)	11.98 (4.45)	9.59 (5.01)	21.78 (9.58)	47.85 (9.74)
5 Undergraduate (bachelor degree)	463 (13.69)						21.49 (3.92)	11.64 (4.27)	9.42 (4.55)	22.23 (10.38)	47.2 (8.5)
6 Postgraduate	533 (15.76)						21.29 (4.09)	11.32 (4.12)	9.01 (4.25)	23.51 (10.7)	46.82 (9.31)
Family status											
1 Not in relationship	1287 (38.05)	*p* < .001 (1–3***, 2–3***)	*p* < .001 (1–2***,2–3***)	*p* < .001 (1–3***, 2–3**)	*p* = .002 (1–2**,1–3*)	*p* < .001 (1–3***, 2–3***)	20.51 (4.62)	12.83 (4.8)	10.91 (5.41)	25.87 (10.88)	46.74 (8.94)
2 In a relationship	1204 (35.6)						20.83 (4.58)	12.74 (4.74)	10.88 (5.29)	24.5 (10.8)	47.75 (8.83)
3 Married	891 (26.35)						21.51 (4.17)	11.33 (3.96)	9.09 (4.24)	24.36 (10.09)	46.28 (8.98)
Gender											
1 Man	1140 (33.71)	*p* = .006	*p* < .001	*p* < .001	*p* < .001	*p* < .001	19.12 (4.74)	11.51 (4.54)	10.06 (4.97)	28.27 (10.59)	45.12 (9.02)
2 Woman	2242 (66.29)						21.79 (4.1)	12.85 (4.59)	10.59 (5.22)	23.33 (10.32)	47.92 (8.73)
Income											
1 up to 30.000 CZK	608 (17.98)					*p* = .019 (1–4**, 1–6*)	20.85 (4.8)	13 (4.84)	10.95 (5.51)	25.71 (10.79)	47.76 (9.47)
2 31.000–40.000 CZK	565 (16.71)						21.14 (4.21)	12.35 (4.55)	10.37 (5.07)	24.33 (10.45)	46.86 (8.57)
3 41.000–50.000 CZK	556 (16.44)						20.89 (4.66)	12.47 (4.59)	10.45 (5.05)	25.23 (10.51)	46.87 (9.26)
4 51.000–60.000 CZK	508 (15.02)						20.94 (4.47)	11.97 (4.38)	10.01 (4.83)	24.68 (10.34)	46.4 (8.86)
5 61.000–70.000 CZK	402 (11.89)						21.08 (4.45)	12.33 (4.48)	10.2 (4.99)	24.75 (10.61)	46.84 (9)
6 71.000 CZK and more	743 (21.97)						20.58 (4.41)	12.22 (4.69)	10.39 (5.22)	25.04 (11.09)	46.98 (8.47)

ODSIS = Overall Depression Severity and Impairment Scale, OASIS = Overall Anxiety Severity and Impairment Scale, TEQ = Toronto Empathy Questionnaire, G_ EOT: Generalized Externally oriented thinking (component of alexithymia), SPSQ_S = Sensory Processing Sensitivity Questionnaire - Sensory Subscale; blank spaces in the columns where group comparison results are shown indicate that no significant differences were found.

### Correlation analysis

Results of the correlation analysis are reported in Table 2. Overall, correlations between variables ranged from low to strong. The strongest relationship was observed between anxiety and depression. Moreover, a negative moderate relationship was found between empathy and externally oriented thinking. Empathy was also weakly positively associated with marital satisfaction, the sensory aspect of the SPS anxiety, and age.

**Table 2:** Correlation table between main study variables.

Variable	1	2	3	4	5	6	7	M	SD
1. TEQ	–							20.89	4.51
2. OASIS	0.08*** (*n* = 3111)	–						12.40	4.62
3. ODSIS	0.02 (*n* = 3104)	0.71*** (*n* = 3104)	–					10.42	5.14
4. G_EOT	− 0.31*** (*n* = 3198)	0.04 (*n* = 3111)	0.07** (*n* = 3104)	–				24.98	10.67
5. SPSQ_S	0.18*** (*n* = 3334)	0.18*** (*n* = 3111)	0.19*** (*n* = 3104)	− 0.12*** (*n* = 3198)	-			46.98	8.93
6. SPSQ_total	0.34*** (*n* = 3334)	0.32*** (*n* = 3111)	0.29*** (*n* = 3104)	− 0.23*** (*n* = 3198)	0.81*** (*n* = 3334)	-		97.55	17.63
7. KMSS	0.13*** (*n* = 2075)	− 0.10*** (*n* = 1933)	− 0.10*** (*n* = 1929)	−0 0.10*** (*n* = 1983)	0.01 (*n* = 2064)	0.01 (*n* = 2064)	-	16.74	4.38
8. Age	0.07** (*n* = 3351)	− 0.20*** (*n* = 3111)	−0 0.20*** (*n* = 3104)	−0 0.06* (*n* = 3198)	− 0.10*** (*n* = 3334)	− 0.12*** (*n* = 3334)	− 0.12*** (*n* = 2095)	31.76	13.13

ODSIS = Overall Depression Severity and Impairment Scale, OASIS = Overall Anxiety Severity and Impairment Scale, TEQ = Toronto Empathy Questionnaire, KMSS = Kansas Marital Satisfaction Scale, G_EOT = Generalised Externally Oriented Thinking (a component of alexithymia), SPSQ_S = Sensory Processing Sensitivity Questionnaire - Sensory Subscale, SPSQ_total = Sensory Processing Sensitivity Questionnaire - Total score, M = mean, SD = standard deviation, * *p* < .05; ** *p* < .01; *** *p* < .001. A zero-order rank Spearman correlation coefficient was used

### Network analysis

#### Anxiety

##### Total score analysis

**Fig. 1:**
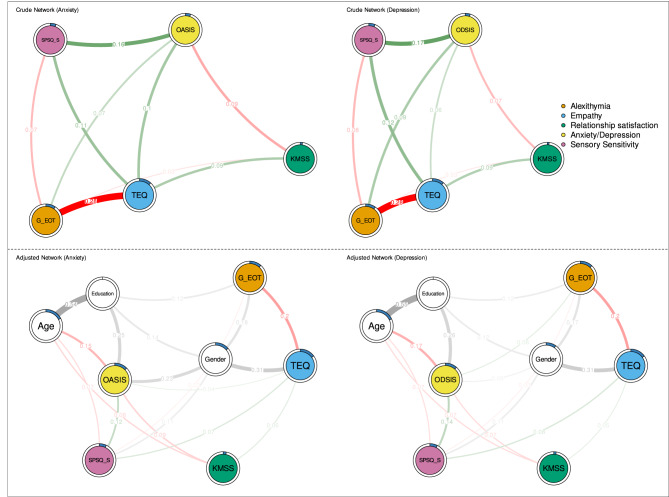
A crude and adjusted network of anxiety and depression - each treated as a total score. Thicker edges represent stronger associations, while thin edges reflect weak associations. Red edges reflect negative associations, green positive associations, and gray undirected significant associations. Rings around the circles refer to node predictability, which exact values can be found in the Supplementary Material (table S1). A higher degree of gray color inside these circles reflects higher node predictability. White nodes represent adjusting variables. ODSIS = Overall Depression Severity and Impairment Scale, OASIS = Overall Anxiety Severity and Impairment Scale, TEQ = Toronto Empathy Questionnaire, KMSS = Kansas Marital Satisfaction Scale, SPSQS = Sensory Processing Sensitivity Questionnaire - Sensory processing sensitivity subscale G_EOT: Generalized Externally oriented thinking (component of alexithymia).

In the adjusted network, empathy was positively linked with SPSQ_S, relationship satisfaction, and anxiety. Moreover, satisfaction in romantic relationships was a node through which empathy was also indirectly linked with anxiety (Fig. 1). Another indirect relationship was observed in the SPSQ_S node through which empathy was linked with anxiety. In this network, two negative links also emerged: first, between romantic relationship satisfaction and anxiety, and second, between alexithymia and empathy. After bootstrapping, the 95% confidence intervals around the edges linking (i) empathy and anxiety and (ii) empathy and relationship satisfaction widened substantially, indicating low precision in the estimated strengths of these associations, although the edges themselves remained present in the network.

In a network without adjusting variables, all relationships were stronger. These links were stable after subsequent bootstrapping. In the adjusted network, the predictability of nodes was highest in age, whereas in the crude network, the highest predictability was achieved in empathy (Fig. 1). The exact values of node predictability and the bootstrapped edge weights can be found in supplementary table S1 and supplementary table S2 respectively.

##### Depression

In both adjusted and crude effect networks of depression, there were several differences as compared with the above-described anxiety network. First, the link between empathy and depression was weak in the crude network (edge weight: 0.05) and disappeared entirely in adjusted network. Second, alexithymia was directly related to depression in the crude but not in the adjusted networks. Third, the link between empathy and relationship satisfaction exhibited low edge‑weight accuracy after bootstrapping; the 95% non‑parametric bootstrap confidence interval around this edge was wide, signalling considerable sampling variability and therefore limited precision in estimating the strength of this association. Other relationships in the depression network were similar to the anxiety network, including node predictability, which was the highest in age (adjusted network) and empathy (crude network) - see Fig. 1. Exact node predictability values and the bootstrapped edge weights can be found in supplementary table S1 and supplementary table S2 respectively.

#### Individual item network

##### Anxiety

**Fig. 2:**
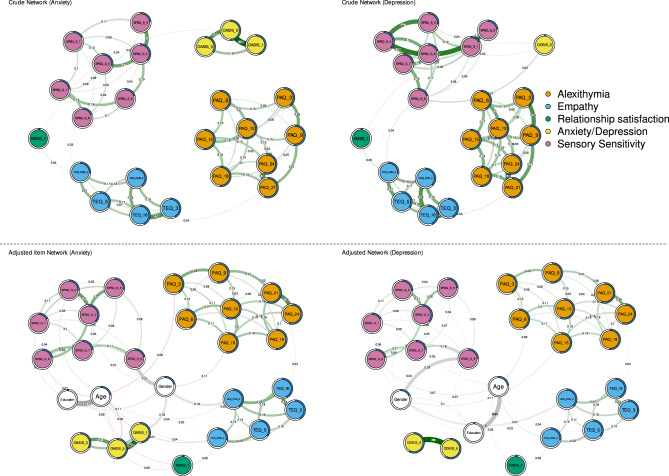
A crude and adjusted network of empathy, alexithymia, sensory aspect of the SPS, relationship satisfaction, and depression items. White nodes represent adjusting variables. ODSIS = Overall Depression Severity and Impairment Scale, OASIS = Overall Anxiety Severity and Impairment Scale, TEQ = Toronto Empathy Questionnaire, KMSS = Kansas Marital Satisfaction Scale, SPSQ_S = Sensory Processing Sensitivity Questionnaire - Sensory subscale, G_ EOT: Generalized Externally oriented thinking (component of alexithymia).

Figure 2 (left side) depicts two networks created from anxiety, relationship satisfaction, sensory aspect of the SPS, alexithymia, and empathy items in both crude and adjusted form. In the adjusted as well as in the crude networks, no negative empathy item was related to anxiety. However, one positive empathy item was indirectly linked with anxiety in the following way: a higher degree of positive empathy (TEQ_CON_4: “When someone close to me is happy, it affects me deeply - in a positive way ”) was related to higher relationship satisfaction (KMSS_1: “How satisfied are you with your partner relationship?”), which was then inversely linked with anxiety (OASIS_5: “How much has your anxiety affected your social life and relationships?”). This indirect link was observed in both crude as well as in adjusted networks. However, after bootstrapping, the accuracy of this indirect link decreased. Interestingly, alexithymia did not have any direct link with anxiety. Nevertheless, there was (in the crude network) an indirect link of alexithymia (PAQ_9: “I don’t pay attention to my emotions”) on anxiety via positive empathy (TEQ_CON_4: “When someone close to me is happy, it affects me deeply (in a positive way ”) and relationship satisfaction (KMSS_1: “How satisfied are you with your partner relationship?”) - see Fig. 2.

##### Depression

In adjusted and crude depression networks (Fig. 2), not a single empathy node was related to depression. Similarly, depression nodes were not associated with any other node in the network except each other (adjusted network – Fig. 2). Relationships between other items in the depression network, e.g., between empathy and alexithymia, remained the same as in the anxiety network. In the adjusted network, the highest predictability was found in depression (ODSIS_5: “How much has your depression affected your social life and relationships?”). In the crude network, the highest node predictability was observed in alexithymia (PAQ_12: “Usually, I try to avoid thinking about what I’m feeling”). In depression network, expected influence was not calculated.

### Expected influence

The expected influence was calculated in the anxiety network, where empathy had an indirect link with anxiety while controlling for adjusting variables. We calculated the expected influence in this network because the link between empathy and anxiety was observed here.

**Fig. 3:**
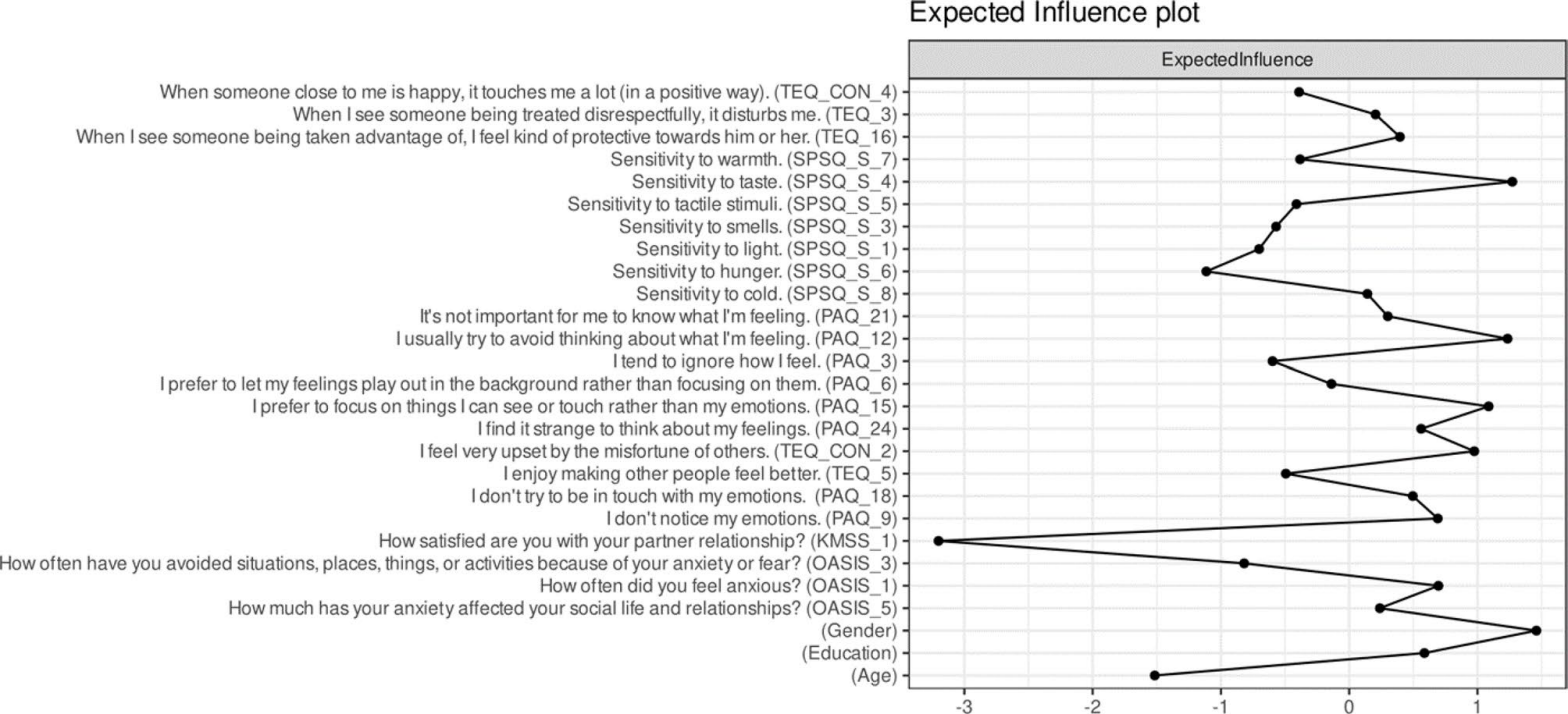
represents the expected influence of nodes in the network (anxiety-adjusted network). The x-axis represents the expected influence quantified in Z-scores - higher values indicate higher expected influence, and lower values lower expected influence. ODSIS = Overall Depression Severity and Impairment Scale, OASIS = Overall Anxiety Severity and Impairment Scale, TEQ = Toronto Empathy Questionnaire, KMSS = Kansas Marital Satisfaction Scale, SPSQ _S = Sensory processing sensitivity subscale of the Sensory Processing Sensitivity Questionnaire, G_ EOT: Generalized Externally oriented thinking (component of alexithymia.

In the anxiety network, the CS-coefficient values (*r* = .75) and the centrality stability plot (Supplementary material: figure S1) suggested that the network is sufficiently stable so that the expected influence index can be interpreted. We found that the node with the highest expected influence in the network was gender, followed by sensitivity to taste (SPSQS_4) and externally oriented thinking (PAQ_12: “Usually, I try to avoid thinking about what I’m feeling”) - Fig. 3. The lowest expected influence was observed in romantic relationship satisfaction (KMSS_1: “How satisfied are you with your partner relationship?“.

### Node differences in expected influence

**Fig. 4:**
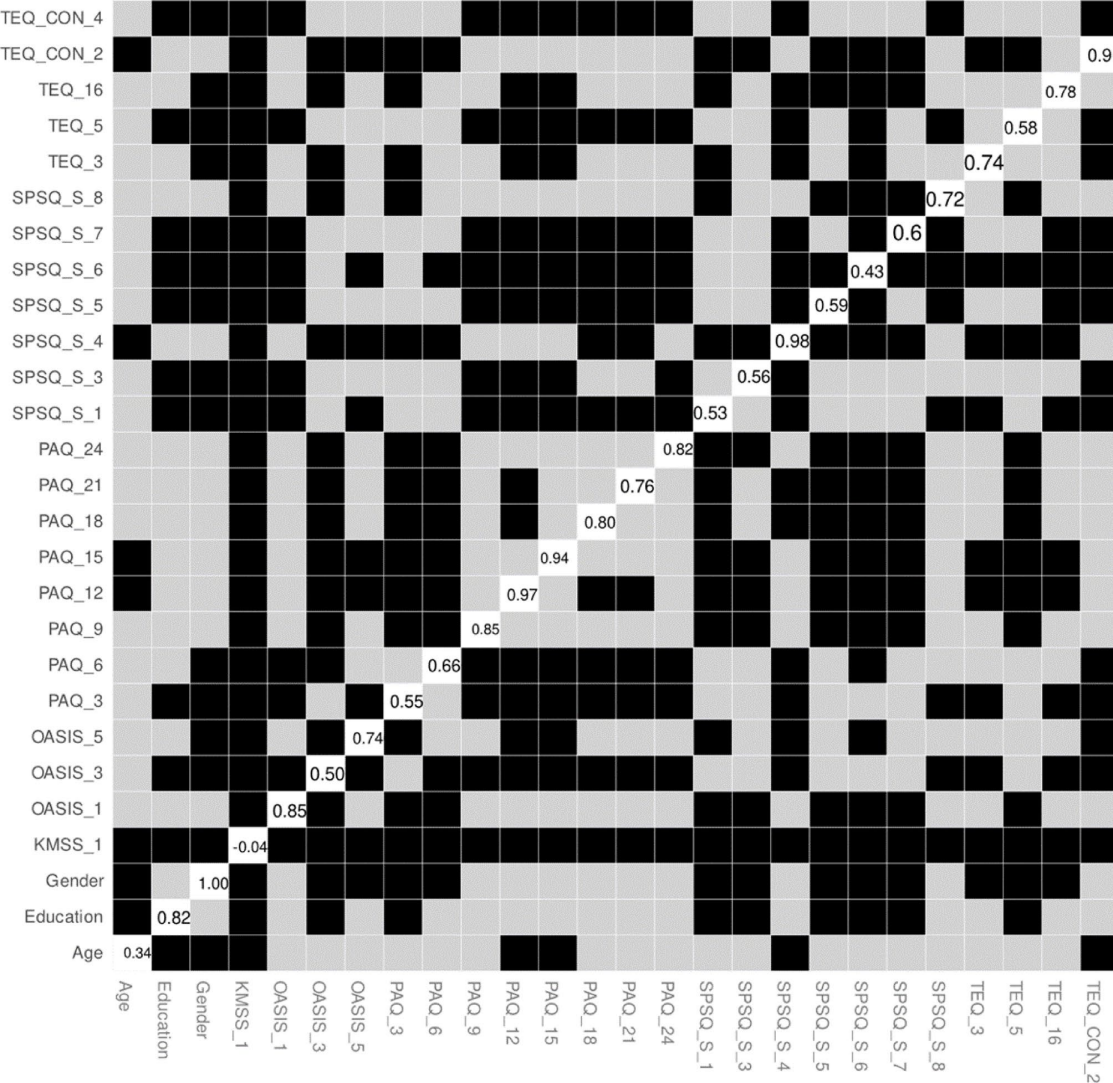
Results of the bootstrapped different test analysis significant differences between nodes in their expected influence. Gray boxes indicate nodes or edges that do not differ significantly from one-another and black boxes represent nodes or edges that do differ significantly from one to another. White boxes in the centrality plot show the value of node expected influence. During bootsrapped difference test, alpha level was set to 0.05.

In the final step, we aimed to test whether nodes in the network are significantly different from each other in the expected influence index. Results of this analysis are presented in Fig. 4. The bootstrapped difference test revealed that the majority of nodes statistically differed from each other in expected influence (black boxes in Fig. 4). Gender was the most distinctive node: its expected influence value significantly differed from most other nodes in the network, including every OASIS item and almost all PAQ, TEQ, and SPSQ_S items. Two further nodes—Sensitivity to taste (SPSQ_S_4) and the externally oriented-thinking item “I usually try to avoid thinking about what I’m feeling” (PAQ_12)—also differed from roughly half of the network. At the opposite extreme, KMSS_1 (“How satisfied are you with your partner relationship?”) recorded the lowest expected influence and was significantly lower than all other nodes in the network. Importantly, because the same nodes were tested multiple times, results need to be interpreted with caution.

## Discussion

The main aim of the present study was to explore the interplay between empathy, anxiety/depression, alexithymia, SPS, and relationship satisfaction. At the level of total scores, emotional empathy was negatively related to alexithymia and positively related to SPS, relationship satisfaction, anxiety, and depression. When focusing on the level of individual items, negative empathy was unrelated to anxiety, but one positive empathy item (“When someone close to me is happy, it affects me deeply (in a positive way).” was indirectly linked to anxiety through higher relationship satisfaction. In the network where anxiety items were replaced by depression items, there was no connection between empathy and depression. After bootstrapping, the accuracy of all links between empathy and anxiety/depression decreased.

Results revealed that emotional empathy was negatively related to alexithymia. This finding aligns with previous studies that used a similar measure of empathy, defined as empathic concern (compassion and warmth towards others in distress), which also found a negative link with alexithymia^46,49,72^. This negative association can be explained by the fact that individuals with a higher degree of alexithymia may have lower emotion regulation^73^, making it difficult for them to manage negative emotions when they see others in distress, and thus harder for them to feel empathic concern^74^. However, when emotional empathy is defined differently, such as personal distress (feeling upset by others’ emotions), the link with alexithymia can be positive^75^.

We found that emotional empathy was positively related to the sensory aspect of the SPS, consistent with previous studies^76,77^, which also reported the association. These findings can be explained by three SPS theories. First, the Theory of Environmental Sensitivity conceptualises SPS as a higher‑order trait comprising deeper cognitive processing, heightened emotional reactivity, enhanced sensory acuity, and ease of overstimulation^33^. Brain‑imaging studies show that highly sensitive individuals display stronger activation of the somatosensory cortex and mirror‑neuron system when viewing others’ emotions^77^, supporting the idea that richer sensory input feeds directly into empathic reaction.

The second framework is the Differential Susceptibility theory, which posits that heightened reactivity amplifies responses to both negative and positive contexts^78^. Daily‑diary studies confirm that highly sensitive persons report larger mood swings—upward as well as downward—following everyday events^79^. This bidirectional plasticity helps explain why our sample’s high‑SPS participants scored higher in total empathy score: they absorb more emotional information in every direction.

The third theory is the Vantage Sensitivity, which assumes that sensitive individuals derive disproportionate benefit from positive experiences such as warm parenting, psychotherapy, or intimacy^80,81^. In relationship contexts that contain supportive cues, their augmented perceptual gate and mirror‑neuron responsivity might intensify affiliative emotions, thereby translating sensitivity into empathic response and ultimately into higher relationship satisfaction. Consistent with this pathway, experimental work demonstrates that empathic concern fully mediates the link between elevated arousability and relationship satisfaction^82^. It is, however, important to note that this positive effect on relationship satisfaction might be strongly affected by the emotion regulation strategy that a highly sensitive person uses^83^.

Taken together, the three frameworks converge on a mechanism in which richer sensory intake → stronger automatic resonance, → greater empathic response. Another explanation for the relationship between emotional empathy and SPS might rest on the effect of a confounding variable, i.e., neuroticism. However, although neuroticism is positively associated with both SPS^52,77^ and empathy^84,85^, this explanation is not likely to hold in the present study for the following reason. Empathy, as measured by the TEQ, represents a construct similar to empathic concern, which was found to be unrelated to neuroticism by past studies^46,48,84^.

Our results also showed that empathy was positively related to relationship satisfaction. This finding aligns with previous research, also finding the positive links between these two constructs^86–88^. One of the possible explanations for this association is that individuals who are better at understanding their partner’s feelings are more likely to handle conflicts using positive social behaviors. This can, in turn, improve the perceived quality of the relationship^88^.

In our study empathy was positively related to anxiety and depression. This aligns with some previous research^89,90^, while contradicting other studies^91,92^. Specifically, for depression, Salo et al. ^93^ found that higher levels of empathic distress were associated with higher depression symptoms, while Zhang et al.^95^ reported that greater emotional empathy (empathic concern) was linked to fewer depressive symptoms. In terms of anxiety, Gambin & Sharp^89^ demonstrated a positive relationship between emotional empathy and anxiety. However, Alvi et al.^94^ did not find any significant association between affect sharing and anxiety.

These contradictory findings can be partially explained *—*just as in the case of alexithymia—by the fact that previous authors have conceptualised and measured empathy in markedly different ways. In particular, emotional empathy can be decomposed into empathic concern (EC; other-oriented compassion) and personal distress (PD; self-oriented aversive arousal). Meta-analytic evidence indicates that PD is positively associated with both depression and anxiety, whereas EC is weakly—or even negatively—related to these internalising outcomes^22^. The TEQ combines items assessing both constructs to form a unidimensional index of emotional empathy^46,93^. Consequently, total scores inevitably conflate PD-driven risk with any protective influence of EC. It is therefore plausible that the positive associations we observed are disproportionately driven by the subset of TEQ items that elicit distress-heavy reactivity (e.g., “Other people’s misfortunes disturb me a great deal”), while the buffering effects of concern-based items are masked. Another explanation is that even when studies share similar definitions and aim to measure the same construct—i.e., empathy—the items in each scale often capture distinct constructs or different aspects of empathy^94,95^. For instance, Nair et al.^23^ found a small positive association between emotional empathy and anxiety using one set of items, whereas Caycho-Rodríguez^96^ found a moderate negative association when measuring the same construct but using different items. Taken together, heterogeneity in item quality and focus could also explain why our results contrast with previous studies.

In the crude effect network, we found that there is a positive relationship between empathy and depression sum scores but this relationship disappeared after controlling for age, gender and education variables. A likely reason the crude total‑score network yielded a weak empathy–depression edge that vanished after adjustment is demographic confounding. Women generally report both higher emotional empathy^97^ and higher rates of depressive symptoms than men^98^; combining scores across genders might therefore induce a spurious positive correlation. Similarly, younger adults show lower empathy^68^ but markedly higher prevalence of major depression compared with older adults^99^ - another pattern that can inflate crude associations. Education works in the same direction: lower educational attainment is linked to higher depression risk^100^, whereas its relationship with empathy is slightly positive^101^. When we controlled for gender, age, and education simultaneously, the overlapping variance responsible for the crude empathy–depression edge was removed, and the association disappeared at both the total‑score and item levels. Taken together, our results suggest that emotional empathy and depressive symptoms are largely independent once demographic variables are taken into account.

Results of analysis focusing on individual items revealed that positive empathy, such as feeling happy when someone close is happy, was indirectly negatively linked to anxiety through higher relationship satisfaction. Specifically, those who felt more positive empathy also reported higher satisfaction in their relationships, which in turn was associated with lower anxiety. This finding is hard to compare with previous studies because this is the first study examining the interactions between these three constructs. Additionally, the explanation of relationships between these variables is not straightforward: while network analysis (unlike traditional methods) might offer an opportunity to generate causal hypotheses between variables^102–105^, it cannot determine the direction of these relationships from cross-sectional data. However, there might be two possible main pathways for causality:

1. Anxiety affects relationship satisfaction, which then influences positive empathy (anxiety → relationship satisfaction → positive empathy).
2. Both anxiety and positive empathy independently influence relationship satisfaction (anxiety → relationship satisfaction ← positive empathy).

When explaining the first pathway, anxiety might impact relationship satisfaction via emotional contagion, meaning that when one partner is anxious, this anxiety is transferred to a spouse^106^. Elevated anxiety levels in both partners could lead to decreased relationship quality. This decline in couple satisfaction may deteriorate emotional bonds between partners, leading to less frequent experiences of feeling happy for a counterpart^107^.

When interpreting the second pathway, the link between anxiety and relationship satisfaction could be explained in the same way as in the first pathway, i.e. via emotional contagion. However, a key difference in this second pathway rests on the link between relationship satisfaction and positive empathy, where empathy is assumed to affect relationship satisfaction. This link can be explained as follows: when people feel joy from their partner’s happiness and express this joy to them, it helps build their emotional capital (i.e., shared positive moments in a relationship), reducing the impact of daily relationship threats, which could consequently lead to increased relationship satisfaction^108^.

In the network where anxiety items were replaced by depression items, there was no connection between emotional empathy and depression. This result cannot be directly compared with previous studies, given that this is the first work examining the relationships between these concepts on the item level. However, this result was unexpected for two reasons: first, many previous studies found an association between emotional empathy and depression^22,90,109^, including a study that used network analysis^110^. Second, anxiety symptoms are usually comorbid with depression symptoms^44,111,112^, and thus, if positive empathy was linked with anxiety (even indirectly), a similar relationship could be expected in depression. The discrepancy between our results and past studies cannot be fully explained by either different conceptualizations or by measurement tools, as we found a relationship between emotional empathy and depression on the level of total scores. One of the possible explanations is that when total scores were deconstructed into individual items, one of these items acted as an adjusting variable and made the relationship between depression and emotional empathy non-significant.

### Strengths and limitations

Our study has several strengths. First, the large sample size allowed us to capture more subtle associations between study variables. Second, we used innovative statistical methods, such as Markov Random Fields and Mixed Graphical Models, which provide a more detailed understanding of the complex interrelationships between variables than traditional methods.

However, our study has some limitations. First, the cross-sectional design limits our ability to establish causal relationships between the variables. Second, the use of snowball sampling and the nature of a sample may affect the external validity of our findings, as bias in participant selection could impact the strength of the relationships observed. For instance, the inclusion of a relatively healthy sample could bias the results, potentially leading to an underestimation or overestimation of the relationships between the studied variables. Third, it is possible that our study did not include relevant variables (e.g., attachment style or childhood trauma) that could affect the link between emotional empathy and anxiety/depression. Fourth, although the two networks were found to be sufficiently stable in terms of centrality indices, bootstrapping of edge weights revealed that edges were unstable overall. This instability means that it is likely that edges were correctly identified, but the exact values of edge weights are uncertain and thus should be interpreted with caution. Finally, the reliance on self-reported data may introduce biases, such as social desirability or recall bias, which could influence the accuracy of the reported findings.

### Implications for practice

Inferring practical implications from the present work is not straightforward. As our study showed that the link between emotional empathy and anxiety is weak, recommendations for practice are problematic to draw. However, our findings provide insights into how biologically-based personality traits, relationship satisfaction, and mood states interact with each other. Therefore, psychotherapists might consider assessing the degree of sensory aspect of the SPS (and possibly the overall SPS) in a client to understand how heightened sensory and emotional responsiveness contributes to anxiety and depression symptoms. By identifying individuals with high sensory aspect of the SPS, therapists can tailor strategies to help manage sensory overload and enhance emotional regulation.

### Implications for research

The findings from this study have several implications for future research. First, since varying conceptualizations of emotional empathy (e.g., empathic concern vs. empathic distress) can lead to different outcomes, it is important for future studies to carefully distinguish between distinct facets of emotional empathy during study design planning, measurement and interpretation of results. Second, given the cross-sectional nature of the present study, it is crucial to extend our findings using experimental design to understand causal relationships in our networks better. Third, researchers should explore networks we fitted using cognitive instead of emotional empathy. This could provide useful insights into how cognitive empathy interacts with anxiety and depression. Last, future investigations should employ SPS instruments that span the full breadth of the trait—while avoiding direct content overlap with empathy items—to disentangle genuine SPS effects from measurement artefacts and to identify which SPS facets exert the greatest influence within affective‑symptom networks.

## Conclusion

Our findings indicate that examining empathy at the individual item level can yield more specific insights into its relationship with anxiety and depression. By deconstructing empathy into its constituent facets, we found a unique association - such as the indirect link between positive empathy and lower anxiety mediated by higher relationship satisfaction. However, these results need to be replicated in the future. We recommend that future research employ longitudinal designs to explore potential causal relationships between specific aspects of empathy and symptoms of anxiety and depression.

## Supplementary Information

Below is the link to the electronic supplementary material.

Supplementary Material 1

## Data Availability

Data, Supplementary materials, programming scripts, and additional resources linked to this research can be accessed through the Open Science Framework (OSF) portal using the specified digital object identifier (DOI) at: 10.17605/OSF.IO/2FHX5.
