# Supplementary material for: Network analysis of empathy, anxiety and depression symptoms, relationship satisfaction, sensory processing sensitivity, and alexithymia
Source: Sci Rep. 2025 Nov 20;15:40935. doi: 10.1038/s41598-025-24677-2 (PMC12635365; doi:10.1038/s41598-025-24677-2)
Supplement: Supplementary file 1 — Supplementary Material 1 [file 41598_2025_24677_MOESM1_ESM.zip › Supplementary_material/Supplementary_tables/table S1.docx]

Supplementary Table S1: Node predictability values across fitted networks

| **Variable** | **total** | | | | **item** | | | |
| --- | --- | --- | --- | --- | --- | --- | --- | --- |
|  | **OASIS** | | **ODSIS** | | **OASIS** | | **ODSIS** | |
|  | **crude** | **adjusted** | **crude** | **adjusted** | **crude** | **adjusted** | **crude** | **adjusted** |
| PAQ_3 |  |  |  |  | 0.397 | 0.407 | 0.400 | 0.411 |
| PAQ_6 |  |  |  |  | 0.422 | 0.422 | 0.423 | 0.423 |
| PAQ_9 |  |  |  |  | 0.521 | 0.521 | 0.522 | 0.522 |
| PAQ_12 |  |  |  |  | 0.566 | 0.566 | 0.559 | 0.559 |
| PAQ_15 |  |  |  |  | 0.518 | 0.524 | 0.517 | 0.523 |
| PAQ_18 |  |  |  |  | 0.509 | 0.509 | 0.509 | 0.508 |
| PAQ_21 |  |  |  |  | 0.556 | 0.556 | 0.555 | 0.555 |
| PAQ_24 |  |  |  |  | 0.544 | 0.544 | 0.544 | 0.544 |
| TEQ_3 |  |  |  |  | 0.415 | 0.415 | 0.415 | 0.415 |
| TEQ_5 |  |  |  |  | 0.343 | 0.351 | 0.345 | 0.351 |
| TEQ_16 |  |  |  |  | 0.429 | 0.430 | 0.429 | 0.430 |
| TEQ_CON_2 |  |  |  |  | 0.387 | 0.392 | 0.371 | 0.379 |
| TEQ_CON_4 |  |  |  |  | 0.261 | 0.281 | 0.265 | 0.275 |
| KMSS_1 |  |  |  |  | 0.021 | 0.030 | 0.018 | 0.026 |
| Age |  | 0.174 |  | 0.179 |  | 0.225 |  | 0.225 |
| Gender |  | 0.135 |  | 0.098 |  | 0.209 |  | 0.176 |
| Education |  | -0.011 |  | -0.006 |  | 0.019 |  | 0.028 |
| OASIS_1 |  |  |  |  | 0.450 | 0.473 |  |  |
| OASIS_3 |  |  |  |  | 0.343 | 0.337 |  |  |
| OASIS_5 |  |  |  |  | 0.496 | 0.504 |  |  |
| SPSQ_S_1 |  |  |  |  | 0.169 | 0.173 | 0.165 | 0.170 |
| SPSQ_S_3 |  |  |  |  | 0.233 | 0.252 | 0.227 | 0.252 |
| SPSQ_S_4 |  |  |  |  | 0.363 | 0.369 | 0.362 | 0.367 |
| SPSQ_S_5 |  |  |  |  | 0.272 | 0.269 | 0.271 | 0.248 |
| SPSQ_S_6 |  |  |  |  | 0.195 | 0.214 | 0.195 | 0.214 |
| SPSQ_S_7 |  |  |  |  | 0.236 | 0.236 | 0.233 | 0.233 |
| SPSQ_S_8 |  |  |  |  | 0.189 | 0.213 | 0.192 | 0.242 |
| G_EOT | 0.095 | 0.116 | 0.099 | 0.121 |  |  |  |  |
| TEQ | 0.117 | 0.150 | 0.114 | 0.150 |  |  |  |  |
| KMSS | 0.022 | 0.031 | 0.018 | 0.024 |  |  |  |  |
| OASIS | 0.050 | 0.126 |  |  |  |  |  |  |
| SPSQ_S | 0.056 | 0.069 | 0.062 | 0.074 |  |  |  |  |
| ODSIS_2 |  |  |  |  |  |  |  | 0.580 |
| ODSIS_5 |  |  |  |  |  |  | 0.030 | 0.585 |
| ODSIS |  |  | 0.048 | 0.115 |  |  |  |  |
| Note. values represent predictability metrics, ODSIS = Overall Depression Severity and Impairment Scale, OASIS = Overall Anxiety Severity and Impairment Scale, TEQ = Toronto Empathy Questionnaire, G_ EOT: Externally oriented thinking (component of alexithymia), KMSS = Kansas Marital Satisfaction Scale, SPSQ_S = Sensory Processing Sensitivity Questionnaire - Sensory Subscale | | | | | | | | |
