# Supplementary material for: Network analysis of empathy, anxiety and depression symptoms, relationship satisfaction, sensory processing sensitivity, and alexithymia
Source: Sci Rep. 2025 Nov 20;15:40935. doi: 10.1038/s41598-025-24677-2 (PMC12635365; doi:10.1038/s41598-025-24677-2)
Supplement: Supplementary file 1 — Supplementary Material 1 [file 41598_2025_24677_MOESM1_ESM.zip › Supplementary_material/Supplementary_tables/table S2.docx]

Supplementary Table S2: Bootstrapped edge weights with 95% confidence internval

| **Edge** | **total** | | | | **item** | | | |
| --- | --- | --- | --- | --- | --- | --- | --- | --- |
|  | **OASIS** | | **ODSIS** | | **OASIS** | | **ODSIS** | |
|  | **crude** | **adjusted** | **crude** | **adjusted** | **crude** | **adjusted** | **crude** | **adjusted** |
| Age--Education |  | 0.66 (0.02,1.06) |  | 0.69 (0,1.1) |  | 0.55 (0.15,0.86) |  | 0.56 (0.15,0.88) |
| Age--OASIS_5 |  |  |  |  |  | -0.06 (-0.17,-0.01) |  |  |
| Age--SPSQ_S_3 |  |  |  |  |  | 0.03 (-0.01,0.11) |  | 0.03 (-0.01,0.12) |
| Age--SPSQ_S_6 |  |  |  |  |  | -0.05 (-0.12,-0.01) |  | -0.05 (-0.12,-0.01) |
| Education--OASIS_5 |  |  |  |  |  | 0.01 (0.01,0.16) |  |  |
| Education--SPSQ_S_1 |  |  |  |  |  | 0.06 (-0.01,0.16) |  | 0.06 (-0.02,0.16) |
| Education--SPSQ_S_3 |  |  |  |  |  | 0.03 (-0.03,0.1) |  | 0.03 (-0.03,0.1) |
| Gender--Education |  | 0.18 (0.01,0.26) |  | 0.15 (-0.02,0.26) |  | 0.15 (-0.02,0.25) |  | 0.12 (-0.06,0.25) |
| Gender--OASIS_1 |  |  |  |  |  | 0.18 (0.11,0.25) |  |  |
| Gender--SPSQ_S_4 |  |  |  |  |  | 0.09 (-0.02,0.17) |  | 0.07 (-0.02,0.18) |
| Gender--SPSQ_S_8 |  |  |  |  |  | 0.34 (0.23,0.39) |  | 0.34 (0.28,0.44) |
| KMSS_1--Age |  |  |  |  |  | -0.05 (-0.12,0.01) |  | -0.04 (-0.12,0.02) |
| KMSS_1--OASIS_5 |  |  |  |  | -0.02 (-0.09,0.02) | -0.02 (-0.11,0.02) |  |  |
| OASIS_1--OASIS_3 |  |  |  |  | 0.18 (0.14,0.24) | 0.18 (0.14,0.23) |  |  |
| OASIS_1--OASIS_5 |  |  |  |  | 0.48 (0.44,0.52) | 0.47 (0.44,0.52) |  |  |
| OASIS_3--OASIS_5 |  |  |  |  | 0.31 (0.28,0.37) | 0.31 (0.27,0.36) |  |  |
| PAQ_12--PAQ_15 |  |  |  |  | 0.19 (0.13,0.25) | 0.19 (0.14,0.25) | 0.19 (0.13,0.25) | 0.19 (0.13,0.25) |
| PAQ_12--PAQ_18 |  |  |  |  | 0.14 (0.08,0.2) | 0.14 (0.08,0.2) | 0.14 (0.08,0.2) | 0.14 (0.08,0.2) |
| PAQ_12--PAQ_21 |  |  |  |  | 0.04 (-0.01,0.1) | 0.04 (-0.01,0.11) | 0.04 (-0.01,0.1) | 0.04 (-0.01,0.1) |
| PAQ_12--PAQ_24 |  |  |  |  | 0.15 (0.1,0.21) | 0.15 (0.09,0.21) | 0.15 (0.09,0.2) | 0.15 (0.09,0.2) |
| PAQ_15--Gender |  |  |  |  |  | 0.09 (0.02,0.2) |  | 0.09 (0.02,0.19) |
| PAQ_15--PAQ_18 |  |  |  |  | 0.18 (0.12,0.24) | 0.18 (0.13,0.24) | 0.18 (0.13,0.24) | 0.18 (0.13,0.24) |
| PAQ_15--PAQ_24 |  |  |  |  | 0.15 (0.1,0.2) | 0.15 (0.1,0.2) | 0.15 (0.1,0.2) | 0.15 (0.1,0.2) |
| PAQ_18--PAQ_21 |  |  |  |  | 0.18 (0.12,0.24) | 0.17 (0.12,0.24) | 0.18 (0.12,0.24) | 0.17 (0.12,0.24) |
| PAQ_18--PAQ_24 |  |  |  |  | 0.1 (0.04,0.16) | 0.1 (0.05,0.16) | 0.1 (0.05,0.16) | 0.1 (0.04,0.16) |
| PAQ_21--PAQ_24 |  |  |  |  | 0.36 (0.3,0.42) | 0.36 (0.3,0.42) | 0.36 (0.3,0.42) | 0.36 (0.3,0.42) |
| PAQ_21--TEQ_5 |  |  |  |  | -0.03 (-0.09,0.01) | -0.03 (-0.09,0.01) | -0.03 (-0.09,0.01) | -0.03 (-0.09,0.01) |
| PAQ_3--Age |  |  |  |  |  | -0.07 (-0.13,-0.02) |  | -0.07 (-0.13,-0.02) |
| PAQ_3--PAQ_12 |  |  |  |  | 0.08 (0.03,0.14) | 0.08 (0.03,0.14) | 0.08 (0.02,0.14) | 0.08 (0.02,0.14) |
| PAQ_3--PAQ_15 |  |  |  |  | 0.1 (0.05,0.15) | 0.1 (0.05,0.16) | 0.1 (0.05,0.15) | 0.1 (0.06,0.16) |
| PAQ_3--PAQ_18 |  |  |  |  | 0.03 (-0.02,0.09) | 0.03 (-0.02,0.09) | 0.03 (-0.02,0.09) | 0.03 (-0.02,0.09) |
| PAQ_3--PAQ_6 |  |  |  |  | 0.15 (0.09,0.2) | 0.14 (0.09,0.2) | 0.15 (0.09,0.2) | 0.14 (0.1,0.2) |
| PAQ_3--PAQ_9 |  |  |  |  | 0.26 (0.2,0.31) | 0.26 (0.2,0.31) | 0.26 (0.21,0.31) | 0.26 (0.21,0.31) |
| PAQ_6--PAQ_12 |  |  |  |  | 0.2 (0.15,0.26) | 0.2 (0.15,0.26) | 0.2 (0.14,0.26) | 0.2 (0.14,0.26) |
| PAQ_6--PAQ_15 |  |  |  |  | 0.16 (0.11,0.22) | 0.16 (0.11,0.22) | 0.16 (0.11,0.22) | 0.16 (0.11,0.22) |
| PAQ_6--PAQ_18 |  |  |  |  | 0.08 (0.03,0.14) | 0.07 (0.03,0.14) | 0.08 (0.03,0.14) | 0.08 (0.03,0.14) |
| PAQ_6--PAQ_9 |  |  |  |  | 0.06 (0,0.12) | 0.06 (0,0.12) | 0.06 (0,0.12) | 0.06 (0,0.12) |
| PAQ_9--PAQ_12 |  |  |  |  | 0.15 (0.09,0.21) | 0.15 (0.09,0.21) | 0.15 (0.09,0.21) | 0.15 (0.09,0.21) |
| PAQ_9--PAQ_15 |  |  |  |  | 0.03 (-0.02,0.08) | 0.03 (-0.02,0.08) | 0.03 (-0.02,0.08) | 0.03 (-0.02,0.08) |
| PAQ_9--PAQ_18 |  |  |  |  | 0.08 (0.02,0.14) | 0.08 (0.03,0.14) | 0.08 (0.03,0.14) | 0.08 (0.03,0.14) |
| PAQ_9--PAQ_21 |  |  |  |  | 0.21 (0.15,0.27) | 0.21 (0.15,0.27) | 0.21 (0.15,0.27) | 0.21 (0.15,0.27) |
| PAQ_9--PAQ_24 |  |  |  |  | 0.05 (-0.01,0.12) | 0.05 (-0.01,0.12) | 0.05 (-0.01,0.12) | 0.05 (-0.01,0.12) |
| SPSQ_S_1--SPSQ_S_3 |  |  |  |  | 0.11 (0.05,0.16) | 0.1 (0.06,0.16) | 0.11 (0.05,0.16) | 0.1 (0.06,0.16) |
| SPSQ_S_1--SPSQ_S_4 |  |  |  |  | 0.08 (0.02,0.14) | 0.08 (0.02,0.14) | 0.08 (0.03,0.14) | 0.08 (0.02,0.14) |
| SPSQ_S_1--SPSQ_S_5 |  |  |  |  | 0.12 (0.06,0.18) | 0.11 (0.06,0.18) | 0.12 (0.06,0.17) | 0.11 (0.05,0.17) |
| SPSQ_S_1--SPSQ_S_7 |  |  |  |  | 0.09 (0.04,0.15) | 0.09 (0.04,0.15) | 0.09 (0.04,0.15) | 0.09 (0.04,0.15) |
| SPSQ_S_1--SPSQ_S_8 |  |  |  |  | 0.06 (0,0.11) | 0.05 (-0.01,0.1) | 0.06 (0,0.11) | 0.05 (0,0.12) |
| SPSQ_S_3--SPSQ_S_4 |  |  |  |  | 0.3 (0.25,0.36) | 0.31 (0.26,0.36) | 0.3 (0.25,0.35) | 0.31 (0.26,0.36) |
| SPSQ_S_3--SPSQ_S_5 |  |  |  |  | 0.04 (-0.01,0.1) | 0.04 (-0.01,0.11) | 0.05 (-0.01,0.1) | 0.04 (-0.02,0.1) |
| SPSQ_S_4--SPSQ_S_5 |  |  |  |  | 0.27 (0.21,0.33) | 0.26 (0.21,0.32) | 0.27 (0.22,0.32) | 0.26 (0.21,0.31) |
| SPSQ_S_4--SPSQ_S_6 |  |  |  |  | 0.19 (0.14,0.24) | 0.19 (0.15,0.25) | 0.2 (0.14,0.24) | 0.19 (0.14,0.25) |
| SPSQ_S_4--SPSQ_S_7 |  |  |  |  | 0.04 (-0.01,0.12) | 0.05 (-0.01,0.12) | 0.04 (-0.01,0.12) | 0.04 (-0.02,0.11) |
| SPSQ_S_5--SPSQ_S_7 |  |  |  |  | 0.08 (0.03,0.13) | 0.07 (0.02,0.13) | 0.08 (0.02,0.14) | 0.07 (0.02,0.13) |
| SPSQ_S_5--SPSQ_S_8 |  |  |  |  | 0.09 (0.04,0.14) | 0.08 (0.03,0.13) | 0.09 (0.04,0.15) | 0.08 (0.03,0.13) |
| SPSQ_S_6--SPSQ_S_7 |  |  |  |  | 0.19 (0.14,0.25) | 0.19 (0.15,0.26) | 0.2 (0.14,0.25) | 0.19 (0.15,0.26) |
| SPSQ_S_6--SPSQ_S_8 |  |  |  |  | 0.11 (0.05,0.16) | 0.11 (0.05,0.16) | 0.11 (0.05,0.17) | 0.11 (0.06,0.17) |
| SPSQ_S_7--SPSQ_S_8 |  |  |  |  | 0.18 (0.12,0.25) | 0.17 (0.11,0.24) | 0.18 (0.12,0.25) | 0.17 (0.12,0.24) |
| TEQ_16--Gender |  |  |  |  |  | 0.02 (-0.03,0.09) |  | 0.01 (-0.02,0.09) |
| TEQ_16--TEQ_CON_2 |  |  |  |  | 0.24 (0.2,0.3) | 0.23 (0.19,0.28) | 0.24 (0.2,0.29) | 0.23 (0.19,0.28) |
| TEQ_3--TEQ_16 |  |  |  |  | 0.33 (0.29,0.38) | 0.33 (0.29,0.38) | 0.33 (0.29,0.38) | 0.33 (0.29,0.38) |
| TEQ_3--TEQ_5 |  |  |  |  | 0.14 (0.1,0.2) | 0.14 (0.1,0.2) | 0.15 (0.1,0.2) | 0.15 (0.1,0.2) |
| TEQ_3--TEQ_CON_2 |  |  |  |  | 0.18 (0.14,0.24) | 0.18 (0.14,0.23) | 0.18 (0.14,0.23) | 0.18 (0.13,0.23) |
| TEQ_3--TEQ_CON_4 |  |  |  |  | 0.06 (0.01,0.12) | 0.06 (0.01,0.13) | 0.06 (0.01,0.13) | 0.06 (0.01,0.13) |
| TEQ_5--Age |  |  |  |  |  | -0.03 (-0.1,0.01) |  | -0.03 (-0.1,0.02) |
| TEQ_5--TEQ_16 |  |  |  |  | 0.17 (0.12,0.22) | 0.17 (0.12,0.23) | 0.17 (0.13,0.23) | 0.17 (0.13,0.23) |
| TEQ_5--TEQ_CON_2 |  |  |  |  | 0.11 (0.07,0.17) | 0.11 (0.06,0.17) | 0.12 (0.06,0.17) | 0.11 (0.06,0.16) |
| TEQ_5--TEQ_CON_4 |  |  |  |  | 0.21 (0.16,0.26) | 0.21 (0.17,0.27) | 0.21 (0.16,0.27) | 0.21 (0.16,0.27) |
| TEQ_CON_2--Age |  |  |  |  |  | 0.03 (-0.02,0.11) |  |  |
| TEQ_CON_2--Gender |  |  |  |  |  | 0.18 (0.1,0.25) |  | 0.19 (0.1,0.26) |
| TEQ_CON_2--TEQ_CON_4 |  |  |  |  | 0.16 (0.12,0.22) | 0.15 (0.1,0.2) | 0.16 (0.11,0.21) | 0.15 (0.1,0.2) |
| TEQ_CON_4--Age |  |  |  |  |  | 0.05 (0,0.14) |  | 0.05 (0,0.13) |
| TEQ_CON_4--Gender |  |  |  |  |  | 0.02 (-0.04,0.09) |  |  |
| TEQ_CON_4--KMSS_1 |  |  |  |  | 0.04 (-0.02,0.1) | 0.04 (-0.01,0.12) | 0.04 (-0.01,0.12) | 0.05 (-0.01,0.13) |
| Age--OASIS |  | -0.15 (-0.2,-0.11) |  |  |  |  |  |  |
| Age--SPSQ_S |  | -0.06 (-0.14,-0.01) |  | -0.06 (-0.14,0) |  |  |  |  |
| Education--OASIS |  | 0.27 (0.04,0.47) |  |  |  |  |  |  |
| G_EOT--Education |  | 0.15 (0,0.24) |  | 0.15 (0,0.23) |  |  |  |  |
| G_EOT--Gender |  | 0.2 (0.09,0.26) |  | 0.19 (0.09,0.25) |  |  |  |  |
| G_EOT--SPSQ_S | -0.06 (-0.14,0) | -0.04 (-0.09,0.03) | -0.07 (-0.14,-0.01) | -0.04 (-0.09,0.02) |  |  |  |  |
| G_EOT--TEQ | -0.27 (-0.32,-0.23) | -0.22 (-0.25,-0.15) | -0.27 (-0.33,-0.23) | -0.22 (-0.25,-0.15) |  |  |  |  |
| Gender--OASIS |  | 0.24 (0.15,0.31) |  |  |  |  |  |  |
| Gender--SPSQ_S |  | 0.1 (0.02,0.19) |  | 0.11 (0.03,0.2) |  |  |  |  |
| KMSS--Age |  | -0.07 (-0.15,-0.01) |  | -0.06 (-0.15,0) |  |  |  |  |
| KMSS--OASIS | -0.08 (-0.16,-0.03) | -0.09 (-0.16,-0.03) |  |  |  |  |  |  |
| OASIS--SPSQ_S | 0.16 (0.11,0.2) | 0.12 (0.08,0.17) |  |  |  |  |  |  |
| TEQ--Gender |  | 0.33 (0.23,0.39) |  | 0.33 (0.23,0.39) |  |  |  |  |
| TEQ--KMSS | 0.08 (0.03,0.15) | 0.07 (0,0.12) | 0.07 (0.01,0.16) | 0.06 (-0.02,0.12) |  |  |  |  |
| TEQ--OASIS | 0.09 (0.04,0.16) | 0.05 (-0.02,0.11) |  |  |  |  |  |  |
| TEQ--SPSQ_S | 0.11 (0.06,0.16) | 0.08 (0.02,0.13) | 0.12 (0.07,0.17) | 0.08 (0.03,0.14) |  |  |  |  |
| SPSQ_S_3--SPSQ_S_8 |  |  |  |  | 0.04 (-0.01,0.09) |  | 0.04 (-0.01,0.1) | 0.03 (-0.01,0.1) |
| TEQ_CON_4--SPSQ_S_8 |  |  |  |  | 0.02 (-0.02,0.08) |  | 0.03 (-0.01,0.09) | 0.01 (-0.01,0.07) |
| G_EOT--KMSS | -0.02 (-0.08,0.02) |  | -0.02 (-0.07,0.02) |  |  |  |  |  |
| G_EOT--OASIS | 0.05 (-0.01,0.14) |  |  |  |  |  |  |  |
| Education--SPSQ_S_8 |  |  |  |  |  |  |  | 0.04 (-0.01,0.14) |
| Gender--SPSQ_S_3 |  |  |  |  |  |  |  | 0.03 (-0.03,0.14) |
| ODSIS_2--ODSIS_5 |  |  |  |  |  |  |  | 0.69 (0.67,0.73) |
| Age--ODSIS |  |  |  | -0.17 (-0.22,-0.13) |  |  |  |  |
| Education--ODSIS |  |  |  | 0.23 (0.14,0.38) |  |  |  |  |
| G_EOT--ODSIS |  |  | 0.08 (0.02,0.16) | 0.06 (-0.01,0.12) |  |  |  |  |
| Gender--ODSIS |  |  |  | 0.07 (-0.03,0.19) |  |  |  |  |
| KMSS--ODSIS |  |  | -0.06 (-0.15,0) | -0.07 (-0.16,0.01) |  |  |  |  |
| ODSIS--SPSQ_S |  |  | 0.17 (0.12,0.22) | 0.14 (0.09,0.19) |  |  |  |  |
| KMSS_1--ODSIS_5 |  |  |  |  |  |  | -0.03 (-0.1,0.03) |  |
| ODSIS_5--SPSQ_S_5 |  |  |  |  |  |  | 0.03 (-0.03,0.09) |  |
| ODSIS_5--SPSQ_S_8 |  |  |  |  |  |  | 0.06 (0.01,0.13) |  |
| PAQ_3--ODSIS_5 |  |  |  |  |  |  | 0.02 (-0.03,0.07) |  |
| TEQ--ODSIS |  |  | 0.05 (-0.01,0.13) |  |  |  |  |  |
| Note: *** p < 0.001, ** p < 0.01, * p < 0.05, Edges with exactly 0 values were removed from the table. Missing values in table mean that edge between two variables was note estimated because one of these edges was not included in a network. ODSIS = Overall Depression Severity and Impairment Scale, OASIS = Overall Anxiety Severity and Impairment Scale, TEQ = Toronto Empathy Questionnaire, G_ EOT: Externally oriented thinking (component of alexithymia), KMSS = Kansas Marital Satisfaction Scale, SPSQ_S = Sensory Processing Sensitivity Questionnaire - Sensory Subscale. | | | | | | | | |
